# Effect of Si on the oxidation reaction of α-Ti(0 0 0 1) surface: *ab initio* molecular dynamics study

**DOI:** 10.1080/14686996.2017.1403273

**Published:** 2017-12-13

**Authors:** Somesh Kr. Bhattacharya, Ryoji Sahara, Kyosuke Ueda, Takayuki Narushima

**Affiliations:** ^a^ Research Center for Structural Materials, National Institute for Materials Science, Tsukuba, Japan; ^b^ Department of Materials Processing, Tohoku University, Sendai, Japan

**Keywords:** Ti alloys, surfaces, oxidation, molecular dynamics, 10 Engineering and Structural materials, 401 1st principle calculations, 212 Surface and interfaces, 106 Metallic materials

## Abstract

We present our *ab initio* molecular dynamics (MD) study of the effect of Si on the oxidation of α-Ti(0** **0** **0** **1) surfaces. We varied the Si concentration in the first layer of the surface from 0 to 25 at.% and the oxygen coverage (*θ*) on the surface was varied up to 1 monolayer (ML). The MD was performed at 300, 600 and 973 K. For *θ* = 0.5 ML, oxygen penetration into the slab was not observed after 16 ps of MD at 973 K while for *θ* > 0.5 ML, oxygen penetration into the Ti slab was observed even at 300 K. From Bader charge analysis, we confirmed the formation of the oxide layer on the surface of the Ti slab. At higher temperatures, the Si atoms diffused from the first layer to the interior of the slab, while the Ti atoms moved from second layer to the first layer. The pair correlation function shows the formation of a disordered Ti-O network during the initial stage of oxidation. Si was found to have a strong influence on the penetration of oxygen in the Ti slab at high temperatures.

## Graphical abstract

**Figure:**
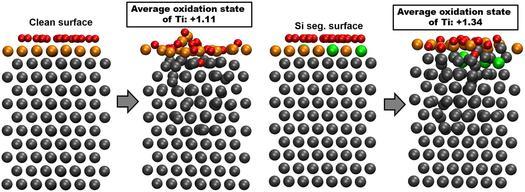

## 1. Introduction

Ti and its alloys are used for high-temperature components such as jet engines and automobile exhaust systems [1,2]. At temperatures above 650 °C, these Ti components, particularly those with thin sections, are deteriorated by reactions with gas oxidants in atmosphere [3]. The oxidation process of Ti involves the growth of outer oxide layer on the surface and the penetration of oxygen into Ti substrate due to high solubility of oxygen in Ti [4–7]. The oxygen rich layer in Ti, which is referred as ‘*α*’ case, causes surface embrittlement and eventually degrades the mechanical properties of the alloys [8]. To improve the oxidation resistance, surface modification processes like surface plating, plasma spraying, ion implantation or physical or chemical vapour deposition are used. However, most of them are expensive and less productive [9–11].

Oxidation of α-Ti alloys has been extensively studied. Stringer [4] and Kofstad [5] reported on the oxidation of commercially pure (CP) Ti in the temperature range of 1073–1473 K and Shih and Jona used low energy electron diffraction (LEED) and Auger electron spectroscopy (AES) to characterize the oxidation of Ti thin plate oriented to Ti(0** **0** **0** **1) at room temperature [12]. Lu et al. studied the oxidation of polycrystalline Ti surface by oxygen and water [13]. Both groups indicated the TiO formation in the early oxidation stage. Kitashima et al. observed that addition of Ge or Sn decreased the oxidation resistance of α-Ti, while Zr, Hf, Si or Nb has beneficial effects [14,15]. Previous experiments have also shown that Si effectively lowers the oxidation rate of Ti [6,16,17]. Chaze and Coddet reported that Si not only prevents the oxide formation on Ti but also the dissolution of oxygen in Ti substrate [6]. Schneider and Ciacchi used molecular dynamics (MD) to characterize the oxidation of Ti films [18]. They performed simulations on microcanonical ensembles and hence, the effect of temperature on the oxidation was not captured. The TiO_2_/Ti interface was studied using density functional theory (DFT) by Ohler et al. [19]. However, the authors considered small system size and short time scale for the MD simulation. Recently, using DFT, we studied the oxygen adsorption on Si-segregated α-Ti(0** **0** **0** **1) surface and the effect of Si on the penetration of oxygen [20]. Si reduces the binding between the oxygen and the surface, while increasing the diffusion barrier for oxygen.

Despite all the works mentioned above, our knowledge on the oxidation of Ti is still limited. From atomistic simulations point of view, little has been done to understand the effects of temperature and oxygen coverage on the oxide layer formation and oxygen penetration in Ti substrate. To the best of our knowledge the effect of Si segregation on the oxidation resistance of Ti has never been studied theoretically. Hence, in this work, we performed *ab initio* MD simulations of the reaction of Si-segregated α-Ti(0** **0** **0** **1) with oxygen at elevated temperatures. Our simulations revealed the effect of Si on the initial stages of the oxide layer formation as well as the penetration of the oxygen into the Ti substrate.

## 2. Computational details

We performed DFT-based MD as implemented in the Vienna *Ab initio* Simulation Package (VASP) [21,22]. The Born–Oppenheimer (BO) approximation was used to decouple the electronic and ionic degrees of freedom. For each BO step, the charge density was optimized by performing non-spin polarized calculation. The valence electron–nuclei interactions were described using the projected augmented wave (PAW) pseudopotentials [23,24], while the Perdew–Burke–Ernzerhof (PBE) [25] exchange-correlation functional was used. The electronic wavefunction was expanded using a plane-wave basis set with an energy cut-off of 500 eV. For the localized augmentation charges, we used a finer mesh of (128 × 128 × 500) for the *x*, *y* and *z* direction, respectively. The Brillouin zone sampling was restricted to the **Г**-point. The Verlet algorithm [26,27] was employed to propagate the ions, and a time step of 0.5 fs was used to integrate the equations of motion. We adsorbed molecular oxygen on the clean and Si-segregated α-Ti(0** **0** **0** **1) surfaces. The Ti(0** **0** **0** **1) surface was modelled using an asymmetric (4 × 4) slab with 11 layers. Further details about the systems are provided in the supplementary information (SI) and the Ti(0** **0** **0** **1) slab model is shown in Figure 1 of SI. The Si was 0–25 at.% (Figure 2 of SI), while the oxygen coverage (*θ*) on the surface was varied within 1 monolayer (ML). We performed canonical ensemble simulations on these systems at 300, 600 and 973 K, and the Nosé thermostat [28] was used to maintain the prescribed temperature. An electrostatic correction was applied along the ***z***-direction of the supercell in order to remove the macroscopic dipole resulting from the oxide formation [29]. We randomly placed oxygen molecules on the clean and Si-segregated Ti(0** **0** **0** **1) surfaces and started the MD simulations. For each system considered and at each temperature mentioned above, we generated a 10 ps trajectory after 8 ps of equilibration.

## 3. Results and discussion

On the clean and Si-segregated Ti surfaces, we adsorbed oxygen molecules following which the MD was started. For the clean and Si-segregated surfaces, we observed the dissociation of oxygen molecules for all *θ* values within first few fs of the MD run at 300 K. The oxygen atoms occupy the threefold coordination sites on the surfaces. The observed spontaneous dissociation of oxygen molecules on the Ti surface is similar to that on Al(1** **1** **1) [30], Si(0** **0** **1) [31] and TiN [32,33].

**Figure 1.**
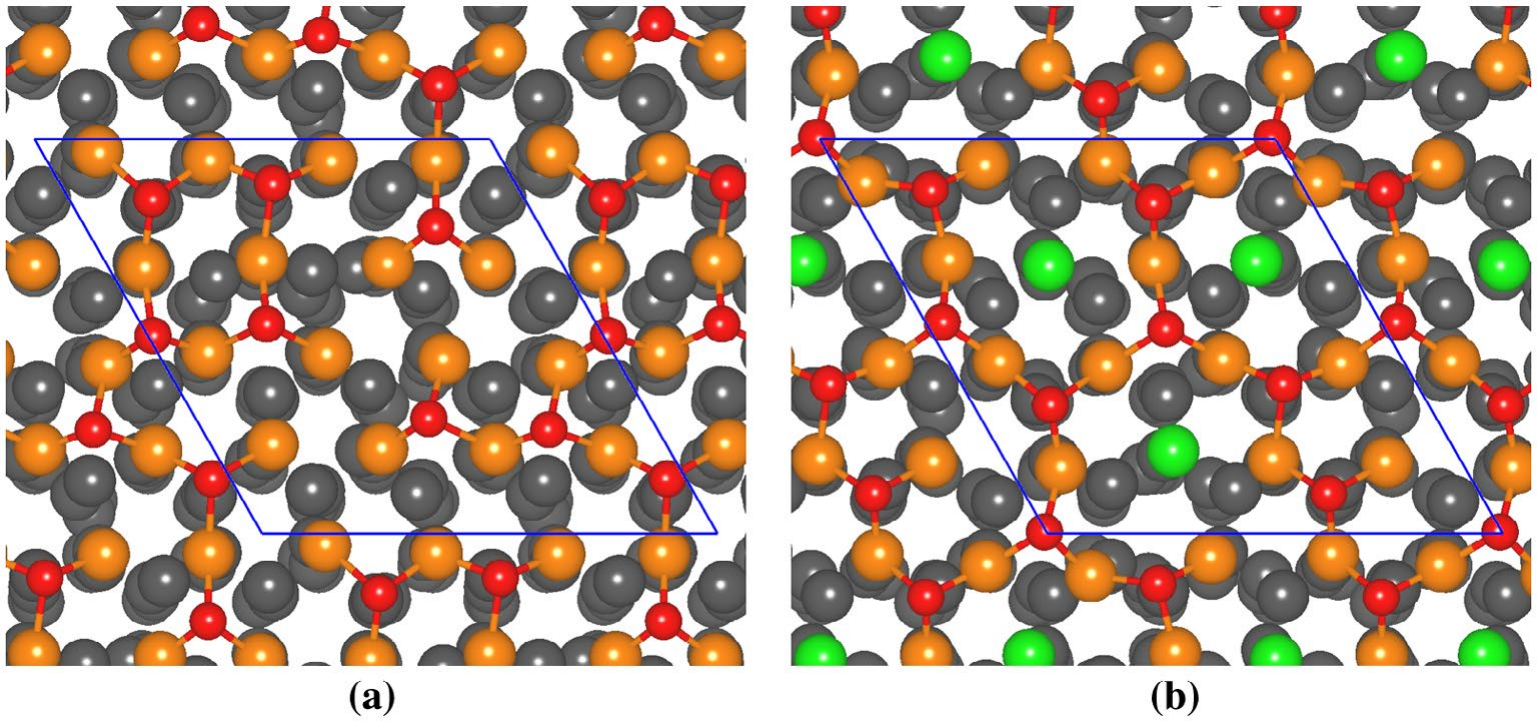
Snapshot (top view) of the (a) clean and (b) Si-segregated Ti(0 0 0 1) surface for theta = 0.5 ML at 973 K. The supercell is repeated along the [0 0 0 1] plane for a better view and the supercell boundary is marked by the solid line. Colour codes: orange – Surface Ti atoms, grey – other Ti atoms, green – Si atoms, red – oxygen atoms.

For *θ* = 0.5 ML, we adsorbed four oxygen molecules on the clean and Si-segregated Ti surfaces. For the clean surface, at 300 K, 75% of the oxygen atoms were observed to occupy the face centred cubic (FCC) sites, while the remaining ones occupied the hexagonal close-packed (HCP) sites. This is due to the fact that the binding between the adsorbate and the adsorbent is stronger at the FCC site compared to the HCP site [20]. No penetration of oxygen atoms into the slab was observed even after 14 ps of dynamics and these oxygen atoms form an ad-layer over the Ti surface as shown in Figure 3(a) of SI. The chemisorption of oxygen on the Ti surface generates the two-dimensional (2D) Ti-O network. When the temperature was raised to 600 K, very little change was observed during the MD as the oxygen atoms remain trapped to their respective FCC or HCP sites. At 973 K, we observed the oxygen atoms to hop between the FCC and HCP sites and the surface of Ti(0** **0** **0** **1) showed a slight reconstruction. Even at 973 K, the penetration of the oxygen atoms into the Ti slab was not observed as shown in Figure 1(a).

At 300 K, the Ti-O network is formed by 8 oxygen atoms and 15 Ti atoms of the first layer. The Bader charge analysis showed that the oxidation state of the surface Ti atoms depends on the coordination of the Ti atoms. Ti atoms with two or three oxygen atoms as their first neighbour (Ti-O distance < 2.5 Å) have an oxidation state of approximately +1, while an oxidation state of ca.+0.5 oxidation state is observed for those with one oxygen atom. Thus, isolated Ti atoms as well as Ti atoms bonded to oxygen atom(s) exist at low temperature. At 973 K, the Ti-O network is formed by 8 oxygen and 16 Ti atoms. The Ti oxidation state obtained from Bader charge analysis is approximately +1 for Ti atoms that have two oxygen atoms as their first neighbour and ca +0.5 for those with only one oxygen atom as their first neighbour. This can be considered as the oxide layer on the Ti slab in which all the Ti atoms of the first layer are bonded to oxygen atoms. The average oxidation state of the Ti atoms in the oxide layer is +0.65 and the oxide layer formed is a disordered network as discussed later.

For Si-segregated case, we considered the Si concentration of 18.75 at.% on the Ti(0** **0** **0** **1) surface. We did not observe oxygen penetration into the slab even after 14 ps of dynamics and the oxygen atoms occupy the surface adsorption sites with minor surface reconstruction as shown in Figure 3(b) of SI. As in case of the clean surface, the 2D Ti-O network is formed on the surface. Only one Si atom forms a bond with the oxygen atom at 300 K. As the temperature is increased to 600 K and finally to 973 K, the surface undergoes slight reconstruction though the oxygen atoms still lie on the surface. Even at 973 K, oxygen did not penetrate into the Ti slab as seen in Figure 1(b). Interestingly, at 973 K, we did not observe any Si-O bonds on the surface. Eight oxygen atoms bonded with the thirteen Ti atoms of the first layer to constitute the oxide layer in which the average oxidation state of the Ti atoms is +0.96 as obtained from the calculated Bader charges. Interestingly, compared to the clean Ti(0** **0** **0** **1) surface, the Ti atoms of the first layer in the Si-segregated surface have a higher oxidation state.

For *θ* = 0.75 ML, six oxygen molecules were adsorbed on the surfaces. For the clean surface, at 300 K and after about 10 ps, one oxygen atom penetrated into the slab and bond with the Ti atoms of the second layer. When the temperature was increased to 600 K and finally to 973 K, the oxygen atom penetrated further into the slab and occupied an octahedral site between the first and the second layers. The remaining oxygen atoms occupied the surface positions and reconstruction of the surface layer was observed. In case of Si-segregated Ti(0** **0** **0** **1) with 18.75 at.% Si, oxygen penetration occurred at 600 K. An interesting observation in this case is the inward diffusion of Si atoms from the first layer to the interior of the slab accompanied by the outward movement of Ti atoms. The Ti atoms move from the second layer towards the first layer. The inward diffusion of the Si atoms resulted in the reconstruction of the surface. The snapshots for the clean and Si-segregated Ti(0** **0** **0** **1) surfaces with *θ* = 0.75 ML at 973 K are shown in Figure 4 of SI.

**Figure 2.**
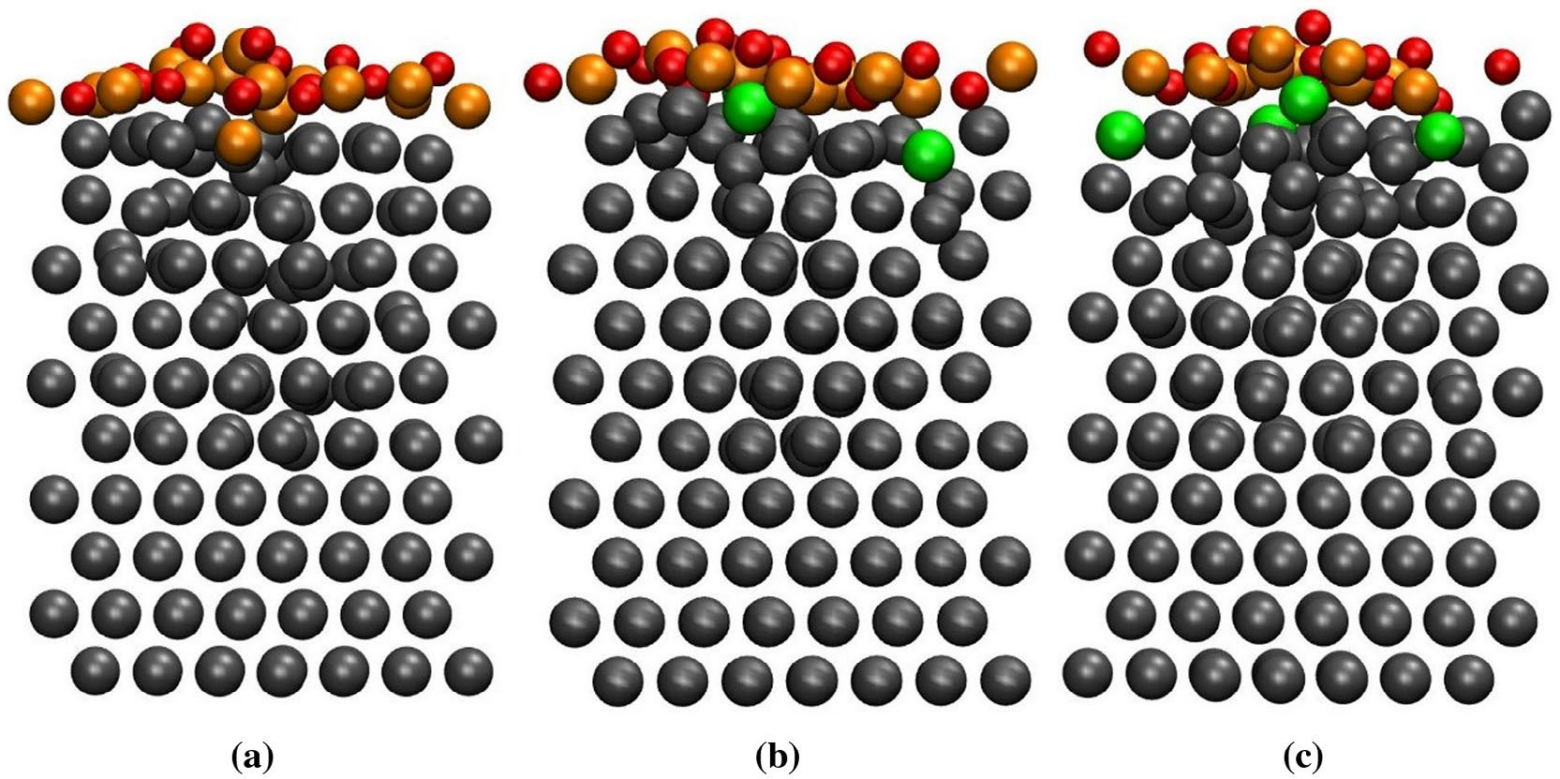
The MD snapshots at 973 K and *θ* = 1.0 ML for the (a) clean surface, (b) 18.75 at.% Si-segregated surface and (c) 25.0 at.% Si-segregated surface. Colour code is the same as in Figure. 1.

For *θ* = 1.0 ML, we adsorbed eight oxygen molecules on the clean and Si-segregated surfaces. For the clean surface at 300 K, one Ti atom from the first layer was moved outward creating an adatom on the surface followed by the penetration of oxygen atoms into the slab. In this case, the surface was reconstructed rapidly compared to lower coverages. When the temperature was increased to 600 K, the oxygen atoms penetrate further and the reconstruction of the surface continued. After 15 ps of dynamics at 973 K, we found one oxygen atom in the second layer, while two Ti atoms of the first layer moved outward. Apart from the first surface layer, the second layer was also reconstructed. A typical MD snapshot for the clean surface at 973 K is shown in Figure 2(a).

In case of the 18.75 at.% Si-segregated surface case, the Ti atoms on the surface were moved outward at 300 K. Moreover, the Si atoms started to diffuse towards the subsurface layers. As the temperature was increased to 600 K, the inward diffusion of the Si atoms continued, while the Ti atoms from the second layer started to move towards the first layer. This is facilitated by the vacancies created in the first layer due to inward movement of the Si atoms. The Si atoms first move from their position in the first layer to interstitial positions between the first and the second layer creating vacancies in the first layer. This is followed by the movement of the Ti atoms from the second layer towards the first layer and finally occupying the vacancy sites in the first layer. The vacancies and interstitials mediated diffusion of atoms is similar to that observed in Si [34]. The penetration of the oxygen atoms into the slab is also observed. Finally, at 973 K, after 15 ps of dynamics two Si atoms diffused and occupied the interstitial space between the second and the third layer (see Figure 2(b)), while one oxygen atom diffused and occupied the octahedral site between the second and the third layer.

In order to understand the effect of Si concentration on the oxidation of Ti surface, we adsorb 1.0 ML oxygen on the 25 at.% Si-segregated Ti surface. At 300 K on this surface, the oxygen molecules dissociated during the early stage of the dynamics. After about 11 ps of dynamics, we observed one oxygen atom at the octahedral site between the first and second layer, while the Si atoms diffused from the first layer to the second layer. At 600 K, the inward diffusion of Si atoms continued, while the surface reconstructed with few more oxygen atoms penetrating into the slab. After 13 ps of dynamics at 600 K, a few Ti atoms were also observed to move outward from the second layer towards the first layer. When the temperature was elevated to 973 K, the outward diffusion of Ti atoms continued from the second layer towards the first layer. The Si atoms moved further inside the slab though further penetration of oxygen atoms into the deeper layers of the slab was not observed even after 18 ps of MD as shown in Figure 2(c).

We analysed the oxidation state of the Ti atoms for *θ* = 1.0 ML using the Bader charges. The oxidation states for the relevant Ti atoms are summarized in Table 1. For the clean Ti(0** **0** **0** **1) surface at 973 K, we confirmed the average oxidation state of the first layer Ti atoms to be +1.11, while the average Ti-O coordination is about four. In the second layer, five Ti atoms have one oxygen each as their first neighbour and have an oxidation state of approximately +0.6. For the 18.75 at.% Si concentration case at 973 K, the Ti atoms of the first layer have an average first-neighbour coordination of four oxygen atoms and an average oxidation state of +1.31 as confirmed from the Bader charges. Few Ti atoms of the second layer and the third layer have one oxygen as their first neighbour and have an oxidation state of approximately +0.5. All these Ti atoms along with the oxygen atoms constitute the oxide layer on the slab. Additionally, the Si atoms were found to have a charged state of approximately −1.5 indicating a charge transfer from the Ti atoms to the Si atoms as the electron affinity of Si is higher than that of Ti. When the Si concentration is increased to 25 at.%, at 973 K the Ti atoms of the first layer have an average Ti-O first-neighbour coordination of four, while their average oxidation state is +1.34. As observed for smaller *θ* values, the average oxidation state of the oxide layer Ti atoms increases with Si segregation. The Si atoms have an oxidation state of approximately −1.5 as observed for the 18.75 at.% Si case.

**Table 1.** The average oxidation state of the Ti atoms of layer ‘1’ for different oxygen coverage and Si concentration are summarized.

Θ (ML)	Si concentration (at.%)	Average oxidation state
0.5	0	+0.65
	18.75	+0.96
0.75	0	+0.88
	18.75	+1.18
1.0	0	+1.11
	18.75	+1.31
	25	+1.34

**Figure 3.**
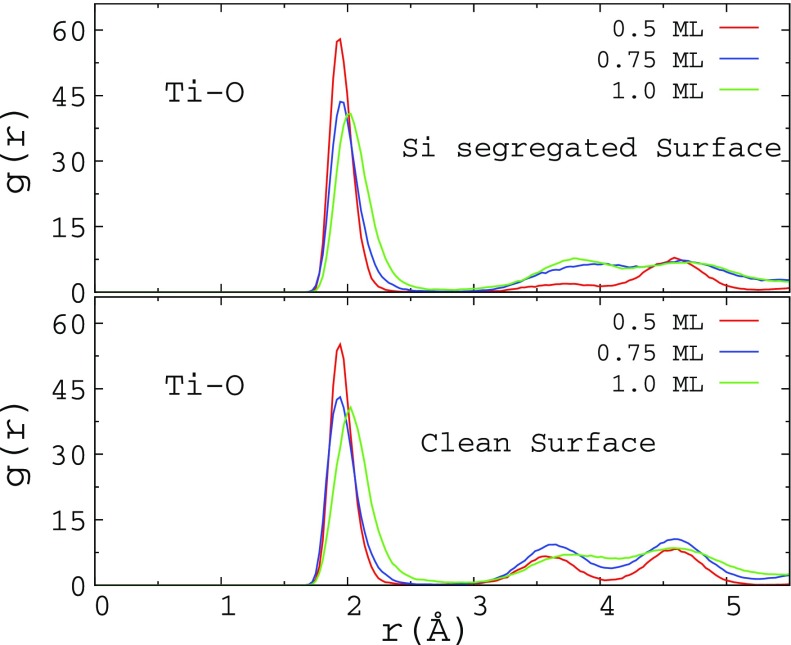
The radial distribution function (*g*(*r*)) for the clean and 18.75 at.% Si-segregated Ti(0 0 0 1) surfaces with different *θ* values at 973 K.

In order to characterize the structural properties of the oxide layer, we calculated the radial distribution function (*g*(*r*)) of these systems. Figure 3 shows the *g*(*r*) for the clean and 18.75 at.% Si-segregated surfaces for various *θ* values at 973 K are shown in Figure 3. From the nature of *g*(*r*), it is clear that during the initial stages of oxidation a disordered network of Ti-O is formed on the clean and Si-segregated Ti surfaces. Lee et al. observed the growth of amorphous oxide layer during the oxidation of Ti films up to 873 K, while crystalline TiO_2_ was observed above that temperature [35]. Schneider and Ciacchi also noted the growth of disordered oxide layer in their MD simulations [18]. For the clean and Si-segregated slabs, the height of the first peak is reduced with increasing *θ*, while the peak width increases. Additionally, the peak height shifts slightly to the right as *θ* changes from 0.75 to 1.0 ML indicating elongation of the Ti-O bonds in the oxide layer.

**Figure 4.**
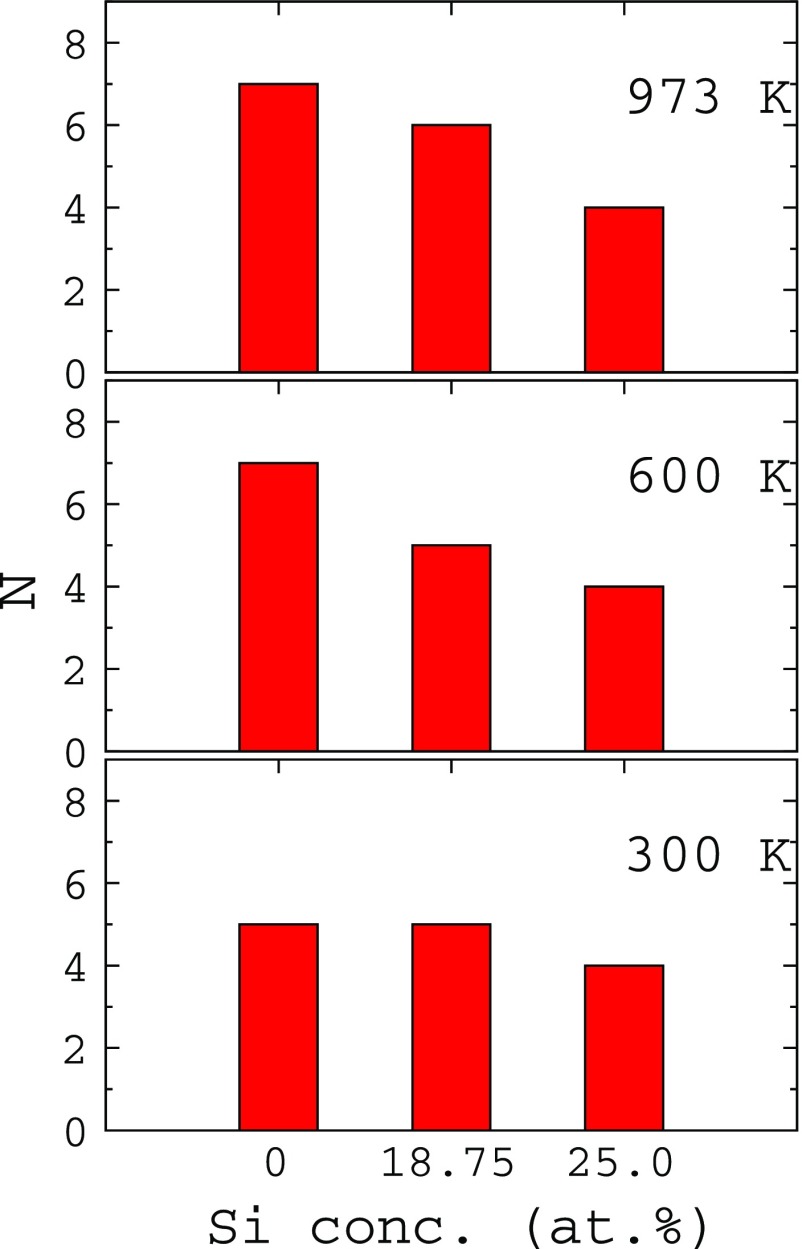
The variation of the number of oxygen atoms (N) in between the first and second layers of the Ti(0* *0* *0* *1) surface with the Si concentration and temperature. The plot is for *θ* = 1.0 ML.

In order to understand the effect of Si on the penetration of oxygen into the Ti slab, we counted the oxygen atoms between the first and second layers at various Si concentrations and temperatures for *θ* = 1.0 ML. The corresponding plot is shown in Figure 4. For all the cases, the number of oxygen atoms penetrating into the slab increases with temperature. However, for a given temperature, as the Si concentration increases the number of oxygen atoms penetrating into the slab steadily decreases. Particularly at 600 K and above, the number of oxygen atoms penetrating the Ti slab with 25 at.% Si segregation is about half of that penetrating the clean surface. This strongly indicates that Si segregation inhibits the penetration of oxygen in the Ti slab and agrees well with the experiments by Chaze and Coddet [6,16]. From the DFT-based calculations, Wu and Trinkle concluded that the diffusivity of oxygen in Ti-Si alloy is reduced compared to pure Ti [36,37]. This result agrees with our MD simulations, where the oxygen penetration is reduced in the presence of Si.

The Si migration from the layer ‘1’ towards the interior of the Ti slab is very intriguing. The calculated Bader charges for the Si-segregated Ti(0** **0** **0** **1) surfaces, prior to oxygen adsorption, show that the Si has a charged state of −1 to −1.5. This shows the tendency to form silicide-like compound on the surface. When this layer comes in contact with oxygen, the Ti atoms seem to undergo preferential oxidation leaving the Si atoms on the surface in a supersaturated state. These Si atoms are thermodynamically unstable as Si concentration >75 at.% at the surface is unstable [20]. Thus, the Si atoms move towards the interior of the slab. In our MD calculations, we observe that the Si migration is directly correlated to the oxidation state of the Ti atoms of layer ‘1’. We calculated the average vertical displacement of the Si atoms along the [0 0 0 1] direction, denoted as $z_{\text{Si}}^{\mathrm{disp}}$ afterwards. For 18.75 at.% Si case, with *θ* = 1.0 ML and at 973 K, the average oxidation state of Ti atoms is +1.31 and the corresponding $z_{\text{Si}}^{\mathrm{disp}}$ is 1.91 Å. For 25 at.% Si, with *θ* = 1.0 ML and at 973 K, the average oxidation state of Ti atoms is +1.34 and the corresponding $z_{\text{Si}}^{\mathrm{disp}}$ is 2.16 Å. This confirms that the higher the oxidation state of Ti, the greater is the migration of Si atoms towards the interior of the slab.

Finally, we would like to comment on the effect of Si on the oxidation resistance of Ti. The oxidation of Ti involves two distinct processes: (i) penetration of oxygen in Ti and (ii) formation of oxide. From our MD simulations, we observed that the presence of Si inhibits the penetration of oxygen into the Ti slab. We calculated the change in the oxidation state of the first layer Ti atoms for the clean and Si-segregated Ti(0 0 0 1) surfaces. For the various oxygen coverages, we found that the oxidation state of the first layer Ti atoms increases by about 25% in presence of Si as shown in Table 1. This indicates the formation of a stable oxide on the surface of Ti and prevent further penetration of oxygen into the interior of the Ti slab consequently enhancing the Ti oxidation resistance.

## 4. Conclusion

We studied the oxidation reactions on clean and Si-segregated Ti(0** **0** **0** **1) surfaces using *ab initio* MD simulations. The oxygen coverage on the surfaces was varied until 1 ML and the oxidation temperature was set to 300, 600 and 973 K. For small coverage, the surface reconstruction is minimal. For *θ* = 0.75 ML, the oxygen penetration into the slab was observed at 300 K, while it is enhanced at 600 and 973 K. For the Si-segregated surfaces, the Si diffusion from the surface layer to the interior of the slab leads to significant reconstruction of the surface and subsurface layers. Simultaneously, the Ti atoms moved from the subsurface layers to the surface. The initial stage of the oxidation of the surface is marked by the formation of a disordered Ti-O network on the metal substrate. The number of oxygen atoms penetrating into the Ti slab reduces as the Si concentration increase. We can conclude that alloying of Ti surfaces with Si will enhance its oxidation resistance as the penetration of oxygen in Ti will be reduced.

## Disclosure statement

No potential conflict of interest was reported by the authors.

## Funding

This work was supported by the Council for Science, Technology and Innovation (CSTI); Cross Ministerial Strategic Innovation Promotion Program (SIP), ‘Process Innovation for Super Heat-resistant Metals (PRISM)’; JST; JSPS KAKENHI [grant number 15H04117].

## Supplemental data

Supplemental data for this article can be accessed here. https://doi.org/10.1080/14686996.2017.1403273

## Supplementary Material

suppl.pdf
