# Supplementary material for: Effect of Si on the oxidation reaction of α-Ti(0 0 0 1) surface: ab initio molecular dynamics study
Source: Sci Technol Adv Mater. 2017 Dec 13;18(1):998–1004. doi: 10.1080/14686996.2017.1403273 (PMC5738634; doi:10.1080/14686996.2017.1403273)
Supplement: suppl.pdf [file TSTA_A_1403273_SM7686.pdf]

Supplementary Information for manuscript "**Effect of Si on the oxidation reaction of α-Ti(0001) surface: *ab initio* molecular dynamics study**"

Somesh Kr. Bhattacharya[*a], Ryoji Sahara[a], Kyosuke Ueda[b] and Takayuki Naushima[b]

[a] Research Center for Structural Materials, National Institute for Materials Science, 1-2-1 Sengen, Tsukuba, Ibaraki 305-0047, Japan. Email: Somesh.Kumar@nims.go.jp

[b] Department of Materials Processing, Tohoku University, 6-6-2 Aza Aoba, Amaraki, Aoba-ku, Sendai 980-8579, Japan.

(A) Ti(0001) surface model and molecular dynamics:

The Ti(0001) surface was modelled using an asymmetric (4x4) slab with 11 layers as shown below.

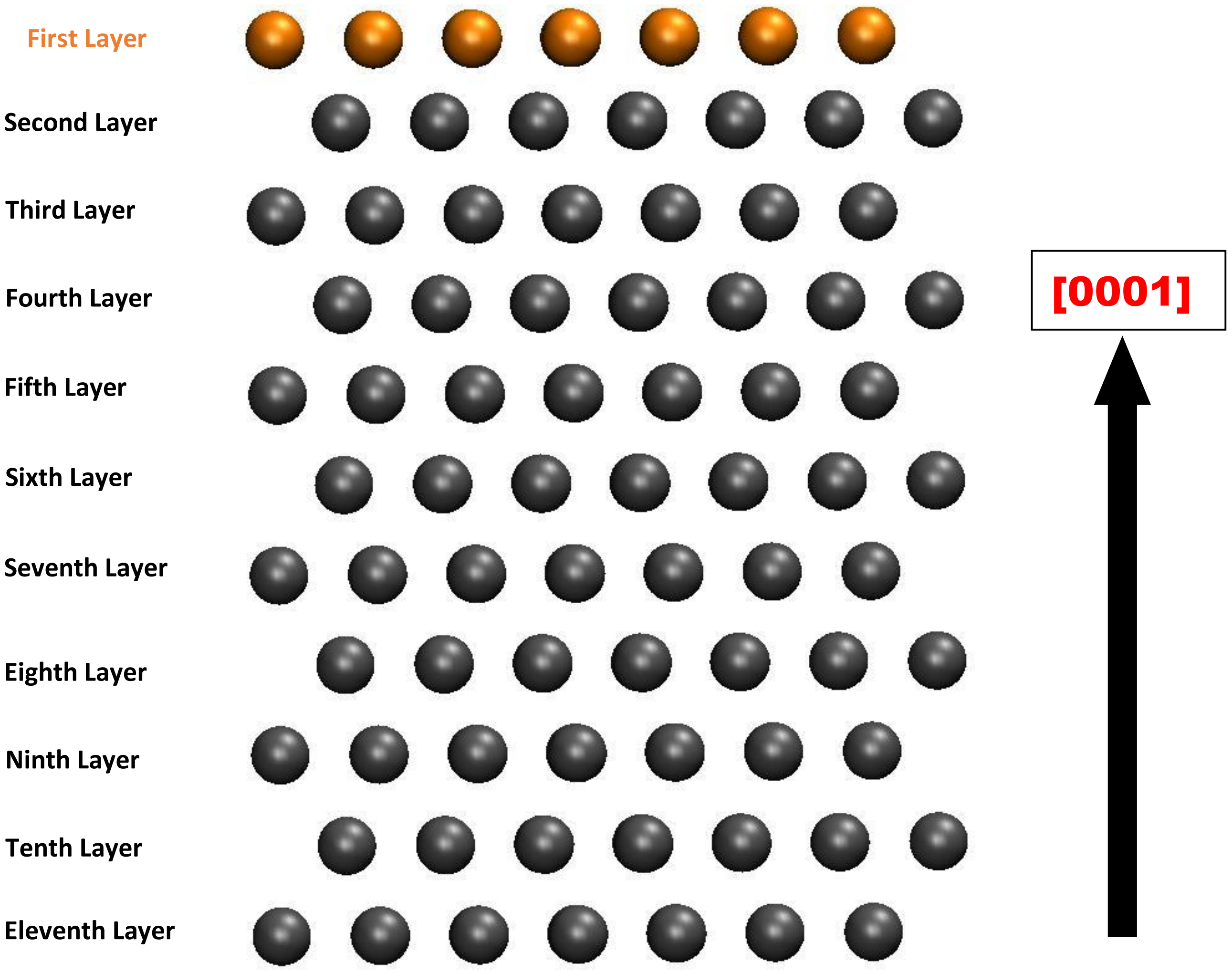

Figure 1. The side view of the Ti(0001) slab model. The layers are marked as First Layer, Second Layer and so on. The first layer is depicted in orange color while other layers are shown in gray.

The bottom four layers, layers 8th - 11th , are kept fixed during the molecular dynaics (MD) while all other layers were allowed to move. The are 16 Ti atoms in each layer and the Ti slab consists of 176 Ti atoms. The Si atoms were substituted at the first layer as shown in our previous work (unpublished paper). To achieve 18.75 at.% and 25 at.% Si concentration, we substituted three and four Si atoms, rspectively, in the Layer 1. The topview for the two different Si segregation cases are shown below.

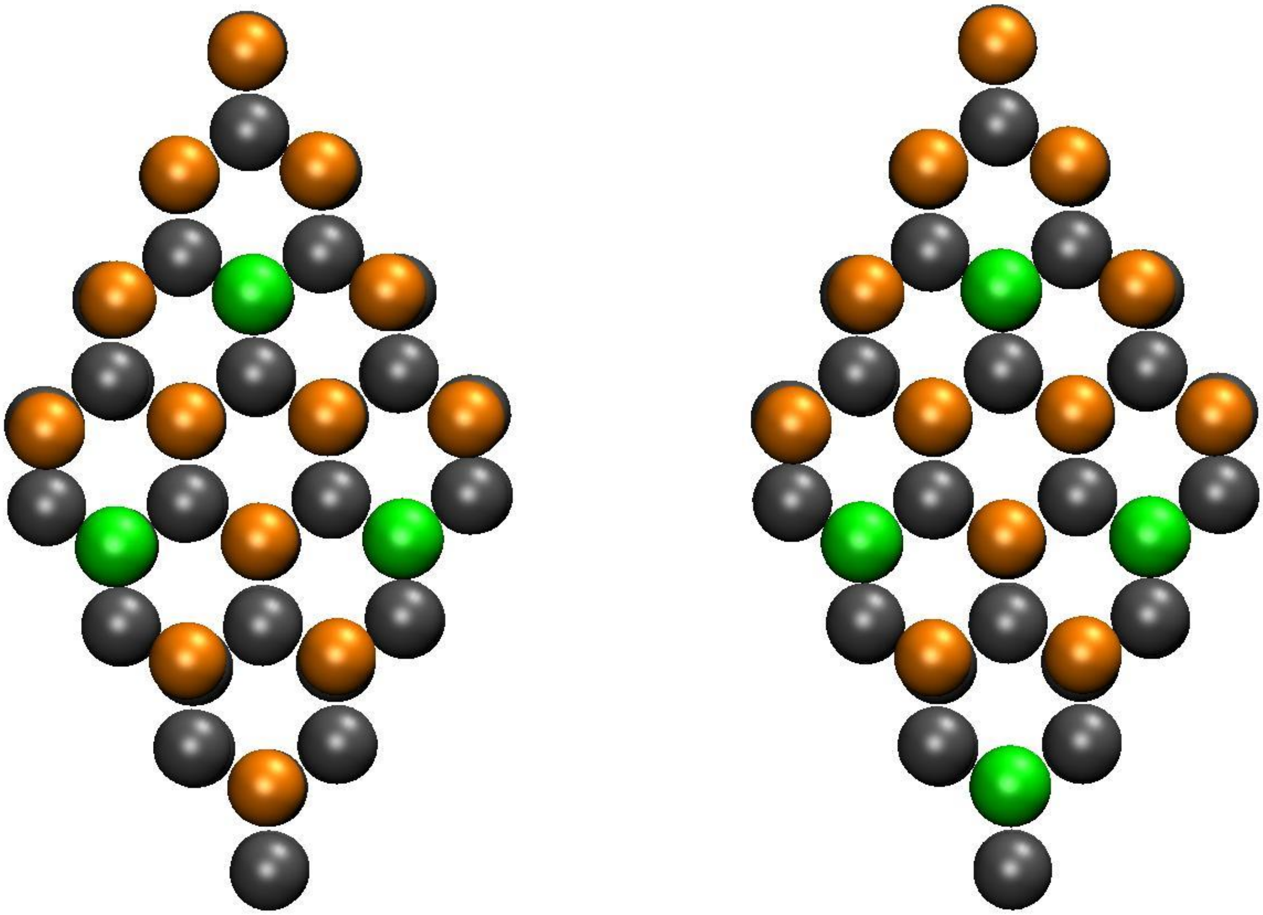

(a) 18.75 at.% Si segregation

(b) 25 at.% Si segregation

Figure 2 Top view of the Si segregated Ti(0001) slab models. Color code: Green- Si atoms, Orange - Ti atoms of First Layer and all other Ti atoms are shown in gray.

Oxygen atoms were adsorbed on the clean and Si segregated Ti(0001) surfaces. The canonical MD was performed at 300K, 600 K and 973 K. We started a microcanonical MD simulation in which the temperature was raised from 0 K to 300 K in 150 steps. We used a time step of 0.5 fs. Upon reaching 300 K, we performed the canonical MD simulation at 300 K. The system was equilibrated for 8 ps and a further 10 ps trajectory was generated for analysis. Once equilibrium was achieved at 300 K, we further raised the temperature to 600 K in 150 steps using the microcanonical MD simulation followed by a canonical simulation at 600 K similar to the 300 K

case. When the system attained equilibrium at 600 K, we finally raised the temperature to 973 K in 373 steps followed by equilibration at 973 K.

(B) **θ = 0.5 ML, T = 300 K**:

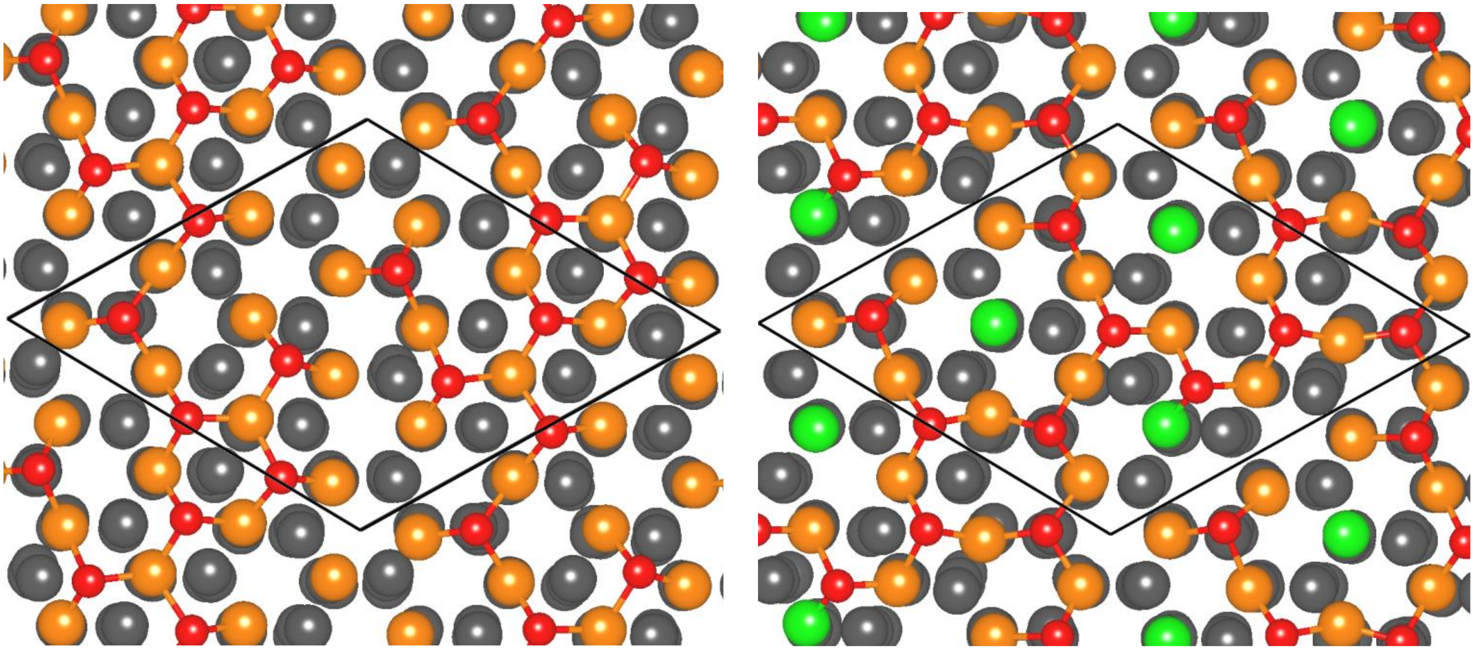

(a) Clean surface **(b) 18.75 at.% Si segregated surface**

Figure 3 Top view showing the Ti-O network for the (a) clean and (b) 18.75 at.% Si segregated surface.

(C) θ = 0.75 ML, T = 973 K

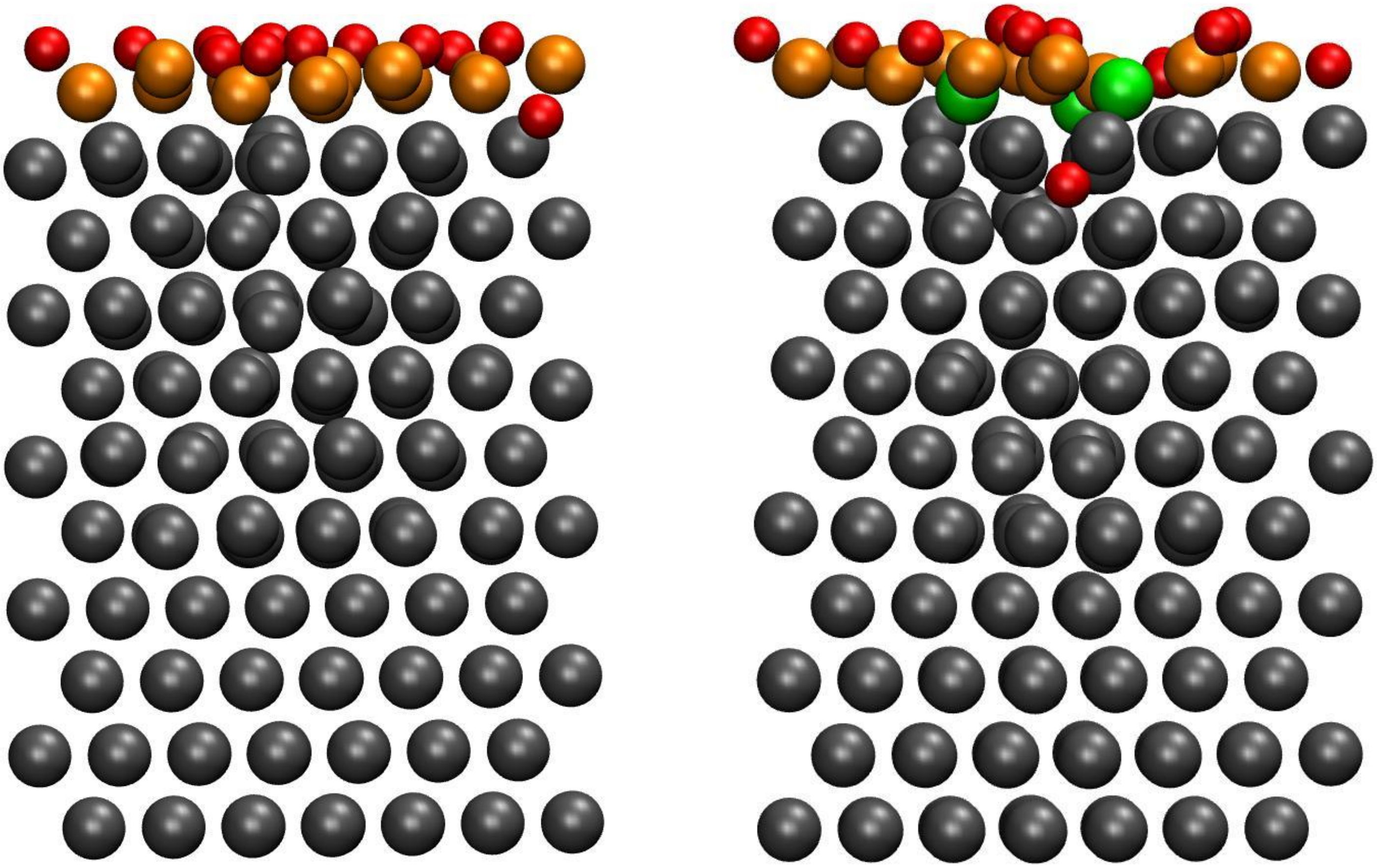

(a) (b)

Figure 4 MD Snapshot for the (a) clean and (b) 18.75 at.% Si segregated surfaces at 973 K with 0.75 ML oxygen.
